# Exploring the associations between physical activity level, cognitive performance, and response to computerized cognitive training among chronic stroke patients

**DOI:** 10.1002/brb3.3406

**Published:** 2024-02-06

**Authors:** Anne‐Marthe Sanders, Geneviève Richard, Knut Kolskår, Kristine M. Ulrichsen, Dag Alnæs, Erlend S. Dørum, Hege Ihle‐Hansen, Mads L. Pedersen, Jan Egil Nordvik, Lars T. Westlye

**Affiliations:** ^1^ NORMENT, Division of Mental Health and Addiction Oslo University Hospital & Institute of Clinical Medicine, University of Oslo Oslo Norway; ^2^ Department of Psychology University of Oslo Oslo Norway; ^3^ Sunnaas Rehabilitation Hospital HT Nesodden Norway; ^4^ Nordre Aasen Foundation Kapellveien Habilitation Centre Oslo Norway; ^5^ Oslo University Hospital Oslo Norway; ^6^ Faculty of Health Sciences Oslo Metropolitan University Oslo Norway; ^7^ Norwegian Directorate of Health Oslo Norway; ^8^ KG Jebsen Center for Neurodevelopmental Disorders University of Oslo Oslo Norway

**Keywords:** cognitive training, physical activity, rehabilitation, stroke, working memory

## Abstract

**Background:**

Post‐stroke attentional and working memory deficits are common and represent relevant predictors of long‐term functional recovery and outcome. The individual responses to cognitive rehabilitation and interventions vary between patients and are influenced by multiple factors. Recently, a link between the level of engagement in physical activities and cognitive rehabilitation has been suggested. However, few previous studies have tested the predictive value of physical activity on cognitive performance and response to cognitive training among chronic stroke patients. There is also a lack of knowledge concerning the prognostic value of index stroke characteristics on physical activity in chronic phase.

**Method:**

In this cross‐sectional and longitudinal study, including stroke survivors suffering mild‐to‐moderate strokes (*n* = 52, mean age = 70 years), we used Bayesian regression to test the association between cognitive performance and response to a 3‐week intervention with a commonly used computerized cognitive training (CCT) system and baseline physical activity level measured with International Physical Activity Questionnaire. We also tested the association between physical activity level in chronic phase and stroke characteristics, including stroke severity (National Institutes of Health Stroke Scale), ischemic stroke etiology (Trial of Org 10172 in Acute Stroke Treatment), and stroke location (*n* = 66, mean age = 68 years). For descriptive purposes, we included 104 sex‐ and age‐matched healthy controls (mean age = 69 years).

**Results:**

The analyses revealed anecdotal evidence of a positive association between overall cognitive performance and daily minutes of sedentary behavior, indicating that better cognitive performance was associated with more daily hours of sitting still. We found no support for an association between cognitive performance and response to CCT with activity level. In addition, the analysis showed group differences in sedentary behavior between patients with small‐vessel disease (*n* = 20) and cardioembolism (*n* = 7), indicating more sedentary behavior in patients with small‐vessel disease. There was no further support for a predictive value of index stroke characteristics on physical activity level.

**Conclusion:**

The results do not support that baseline physical activity level is a relevant predictor of the overall performance or response to CCT in this sample of chronic stroke patients. Similarly, the analyses revealed little evidence for an association between index stroke characteristics and future activity level in patients surviving mild‐to‐moderate stroke.

## Graphical Abstract

Our findings do not support that baseline physical activity level is a relevant predictor of the overall performance or response to computerized cognitive training in this sample of chronic stroke patients. Additional analyses revealed little evidence for an association between index stroke characteristics and future activity level in patients surviving mild‐to‐moderate stroke.

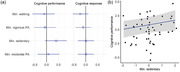

## 1 INTRODUCTION

Stroke is one of the leading causes of death worldwide, and a major cause of short‐ and long‐term cognitive impairments and dementia (Douiri et al., 2013; Krishnamurthi et al., 2020; Leys et al., 2005; Tatemichi et al., 1994). The severity of cognitive impairment, often characterized by working memory and attentional control deficits as well as reduced processing speed (Barker‐Collo et al., 2010; Hochstenbach et al., 1998), may be dependent on the location and severity of the stroke (Sun et al., 2014). Importantly, cognitive deficits post‐stroke may also vary with age (Gorelick et al., 2011), education, occupation (Wu et al., 2013), vascular factors (Sahathevan et al., 2012), and overall brain health as indicated by brain age (Aamodt et al., 2023). Attentional control and working memory enable both the detection, filtering, and temporary storage of relevant information while using that information to perform a cognitive task (Baddeley, 1986), which are key functions to support daily activities (Ponsford et al., 2014). Cognitive disabilities, including attentional deficits, can have major impact on everyday activities, independence (Barker‐Collo et al., 2010), and quality of life (McDowd et al., 2003) and are common focus in stroke rehabilitation (McDonald et al., 2019).

Usually in conjunction with other therapist‐administered approaches, computerized cognitive training (CCT) is used in the rehabilitation of attentional and working memory functions after brain injury (Björkdahl et al., 2013; Bogdanova et al., 2016; Cicerone et al., 2019). Stroke survivors completing 20 days with CCT using Cogmed TM (Cogmed Systems AB) improved working‐memory performance and self‐reported everyday functioning compared to waiting‐list controls (Peers et al., 2020). Overall, however, studies on the effect of CCT on cognitive abilities have been inconclusive, mostly reflected in better performance on attentional tests with similarities to the trained tasks (Johansson & Tornmalm, 2012; Nyberg et al., 2018; Peers et al., 2020; Richard, Petersen, et al., 2020), but likely partly reflecting substantial individual differences in treatment response. The clinical effect of CCT has been shown to vary as a function of various moderating factors, including cognitive abilities (Karbach et al., 2017). Although moderate‐to‐vigorous physical activity has been suggested to enhance post‐stroke cognitive function, especially attention and processing speed (Oberlin et al., 2017), it is unclear if patients with a higher level of baseline physical activity show better cognitive performance and more cognitive improvement in response to CCT compared to patients with a more sedentary lifestyle.

Physical activity is defined as moving the body by the use of skeletal muscles, resulting in energy expenditure (Lee et al., 2019), of which intensity can be categorized as light (e.g., walking), moderate (e.g., brisk walking), or vigorous (e.g., running or jogging), reflecting different levels of energy expenditure (Piercy et al., 2018). Previous studies have demonstrated associations between physical activity of both light and moderate‐to‐high intensity and brain vasculature (Sanders et al., 2023) and brain morphometry (Burzynska et al., 2014; Erickson et al., 2014; Sanders et al., 2021; Tian et al., 2015) in healthy adults. In addition to the positive association between physical activity and cognition among stroke patients (Oberlin et al., 2017), an umbrella review demonstrated moderate evidence from randomized controlled trials of positive associations between cognitive function and moderate‐to‐high intensity physical activity in adults above 50 years of age (Erickson et al., 2019). The conclusion regarding light‐intensive physical activity was not clear due to a lack of studies, but a potential negative effect of sedentary behavior on cognitive function has been suggested in healthy older adults (Falck et al., 2017). Identifying actionable factors for predicting and improving treatment response has clinical relevance, and level of physical activity represents a relevant candidate.

To further understand the potential and role of physical activity in cognitive rehabilitation, more information about the factors influencing physical activity level in chronic phase after stroke is needed. Post‐stroke physical activity is a complex and multifactorial trait associated with age and sex, but also modifiable factors such as fatigue (Thilarajah et al., 2018), cardiorespiratory fitness, depression (Baert et al., 2012), balance (Alzahrani et al., 2012), as well as fall‐related self‐efficacy, physical function, and health‐related quality of life (Vahlberg et al., 2013). Despite a general consensus that these factors influence physical activity, less is known about the prognostic value of index stroke characteristics on physical activity in chronic phase (Thilarajah et al., 2018), including factors related to the location of the stroke, stroke severity, and ischemic stroke etiology. One study, including data from 30 (1 year post‐stroke) and 44 (2 years post‐stroke) patients, reported that patients with right hemisphere infarcts showed higher physical activity level compared to patients with left side strokes (Kunkel et al., 2015). Further, one previous study, including 110 stroke survivors with mild neurological symptoms, defined as a National Institutes of Health Stroke Scale (NIHSS) ≤3 at acute care, reported no significant association between stroke severity and physical activity 6 months after stroke (Wolf & Koster, 2013). Importantly, most studies investigating associations between stroke severity (NIHSS) and physical activity in the chronic phase assessed NIHSS at study entry, not from the acute phase. Studies utilizing NIHSS obtained during the index hospital stay are therefore needed to better inform and improve the multifactorial perspective of potential influencers on physical activity in chronic phase.

To this end, the main aim of the present study was to test the association between physical activity level and overall performance and response to CCT intervention among chronic stroke patients (>6 months post‐stroke). Based on the notion that higher levels of physical activity are associated with better post‐stroke cognitive function, we hypothesized that (1) the patients with higher activity level, both light and moderate‐to‐vigorous physical activity intensity, or a lower number of daily minutes of sitting sill, would show better cognitive performance and more positive effects of the CCT intervention. Secondary, we wanted to assess if index stroke characteristics assessed in the acute phase, including stroke location, ischemic stroke etiology, and stroke severity, could predict activity level in chronic phase (>6 months post‐stroke). Based on previous findings showing physical activity to be multifactorial and associated with multiple health‐related factors, we hypothesized that (2) more severe neurological symptoms in the acute phase would be associated with less physical activity across various intensities and a higher number of daily minutes of sitting still. Exploratory analysis was conducted with regard to the location of the stroke lesion, ischemic stroke etiology, and their association with physical activity level.

We tested the hypotheses using data obtained from chronic stroke patients invited to participate in a longitudinal double baseline study investigating predictors and moderators of cognitive training outcomes, as well as a cross‐sectional healthy control cohort. The sample has previously been described in detail (Kolskår et al., 2021; Richard, Kolskår, et al., 2021; Richard, Petersen, et al., 2020; Ulrichsen et al., 2020). In the current study, cross‐sectional data obtained from 66 stroke patients at first assessment, together with 104 sex‐ and age‐matched healthy controls, and longitudinal data on 52 stroke patients who completed the CCT training, were included in the analysis.

## 2 MATERIALS AND METHODS

### 2.1 Participants

#### 2.1.1 Stroke patients

**FIGURE 1:**
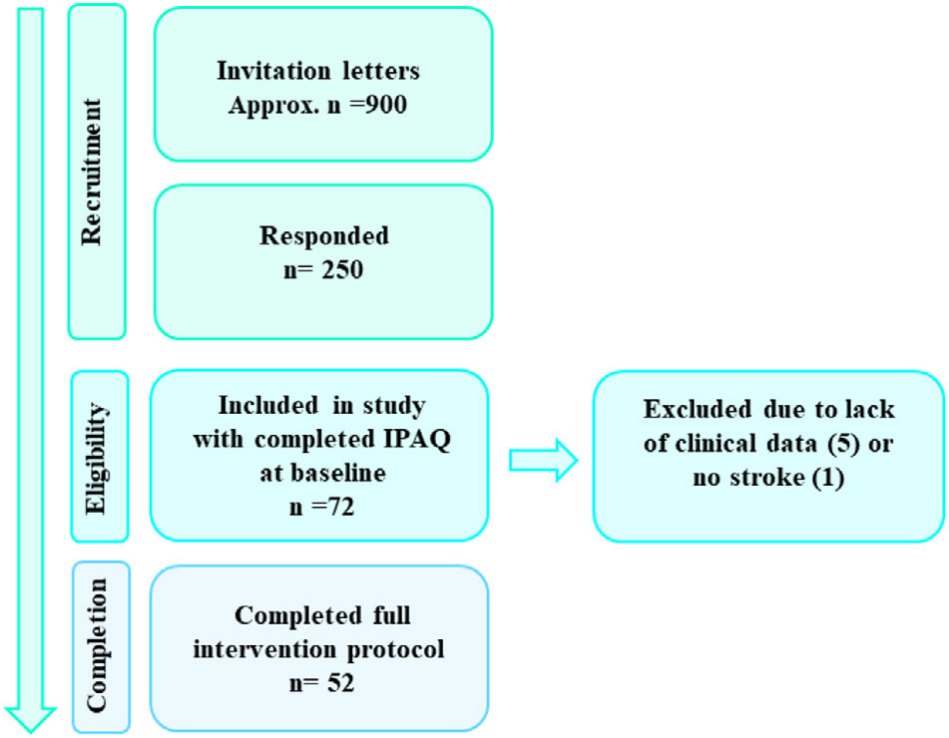
Flow diagram of the inclusion process for stroke patients.

Figure 1 illustrates the inclusion process. Seventy‐six stroke survivors in chronic phase (>6 months since admission), including both ischemic and hemorrhagic strokes, were recruited among previously admitted patients to the Stroke unit at Oslo University Hospital or the Geriatric department at Diakonhjemmet Hospital, Oslo, Norway. Exclusion criteria included transitory ischemic attack, other neurological diseases diagnosed prior to the stroke, severe psychiatric diagnoses, alcohol or drug abuse, and/or MRI contraindications. Details have previously been described (Kolskår et al., 2021). Briefly, after initial screening by phone of participants that responded to invitation, 77 patients were included in the project, of whom 72 completed neuropsychological tests and questionnaires, including physical activity, at baseline. Due to lack of clinical data from stroke onset, five patients were excluded from the current study, and one was excluded due to lack of confirmed stroke. Of this sample, 52 participants completed the 17 CCT sessions. Drop‐out from the study was partly due to high workload of the intervention. All participants were ambulatory at inclusion. In addition, the participants did not report severe visual deficits or aphasia and scored a median of 1 (IQR = 2, min–max = 0–7) on the NIHSS at discharge from acute hospital after the stroke, representing mild‐to‐moderate strokes (Hinkle, 2014). All participants provided written consent at inclusion and were compensated for their time (500 NOK). The study was conducted according to the Helsinki Declaration and approved by the Regional Committee for Medical and Health Research Ethics South‐East Norway (2014/694, 2015/1282).

#### 2.1.2 Healthy controls

Healthy controls were included in the current study due to descriptive purposes. Recruitment was done through advertisements in local papers, social media, and word of mouth, and candidates were screened for eligibility (Dørum et al., 2020; Richard et al., 2018). Exclusion criteria included neurological disorders such as stroke, traumatic brain injury or dementia, history of severe psychiatric diseases, medications significantly affecting the nervous system, abuse of alcohol or other drugs, and/or contraindications for MRI. From the pool of 341 healthy participants (age = 20–94, mean [SD] = 59 [14.8], female = 62%), we extracted 104 participants matched for age and sex with the patient sample at the group level (age = 48–82, mean [SD] = 69 [7.1], female = 25%) using the R package *Matchit* and the method *nearest* (Ho et al., 2011) with a 2:1 ratio.

### 2.2 Cognitive intervention

#### 2.2.1 Cogmed

CCT was applied using Cogmed TM (Cogmed Systems AB), as previously described (Kolskår et al., 2021; Richard, Petersen, et al., 2020). Briefly, Cogmed is a cognitive intervention, primarily targeting visuospatial and verbal working memory functions. The current implementation was internet‐based, allowing the patients to practice at home, and adaptive, with an active reward and feedback system, including sound and points, designed to maintain task engagement (Westerberg et al., 2007). The participants completed 17 sessions over 3–4 weeks, with 8 tasks over 45 min per session, and approximately 5 sessions per week. The training was both home‐ and hospital‐based, with two of the weekly sessions performed at the hospital.

### 2.3 Physical activity

The Norwegian translation of International Physical Activity Questionnaire ([IPAQ] IPAQ, 2005) was used to assess the duration of weekly physical activity in stroke survivors and healthy controls. This is a seven‐item questionnaire, asking for the number of days and duration (minutes and hours) of vigorous (running), moderate (fast walking), and low (walking) intensity activity, in addition to daily sedentary minutes (sitting). The registered activities had a minimum duration of 10 min. The registration of data was conducted according to published guidelines, including the truncation of values within each category of intensity exceeding 180 min/day (IPAQ, 2005). The data was added up to calculate the total amount of time (minutes/week) within each intensity. Sedentary time was measured as time spent sitting or lying down in all waking hours during a typical day. Trained health professionals assisted at first meeting to clarify any questions from the participants regarding the questionnaire. Measurement properties have previously been reported (Craig et al., 2003).

### 2.4 Clinical measures

Ischemic strokes were classified according to the Trial of Org 10172 in Acute Stroke Treatment (TOAST) according to etiology, within the categories of large‐artery atherosclerosis, cardioembolism, small‐vessel disease, stroke of another determined cause, and stroke of an undetermined cause (Adams et al., 1993). In the analysis of differences between subtypes of stroke and physical activity level, the participants categorized as “other” or “undetermined” were excluded. Location of stroke lesions was categorized into four categories, left‐ and right‐hemispheres, bilateral, or cerebellum/brainstem. Severity of neurological symptoms was measured according to the NIHSS at discharge from acute hospital, with more severe neurological symptoms indicated by a higher score (Hinkle, 2014; American Academy of CME, 2021). NIHSS is a widely used clinical tool with predictive value for motor recovery after stroke (Stinear et al., 2017). Cognitive impairment was assessed using the Montreal Cognitive Assessment, which has high specificity and sensitivity (Pendlebury et al., 2010) and has proven accuracy for neurocognitive difficulties among stroke patients (Munthe‐Kaas et al., 2021).

### 2.5 Processing of CCT results

The calculation of CCT performance and improvement scores was completed as previously described (Kolskår et al., 2021; Richard, Petersen, et al., 2020). Briefly, each participant's performance across both sessions and tasks was extracted, and average performance across sessions for each task was used as a marker for average working memory capacity within each CCT task. By estimating a linear model for each participant and task across sessions, derived beta‐values for each model were utilized as a proxy for training improvement within each task across the intervention. Zero‐centered and standardized coefficients from the linear models as well as mean performance were separately submitted to a principal component analysis (PCA). The first factor from the beta‐estimate PCA was used as a proxy for individual improvement in performance across all CCT tasks, whereas the first factor from the mean performance PCA was utilized as a proxy for mean working memory capacity. Due to many outliers, the subtasks “Hidden objects” and “Digits” were excluded from all analyses. Six Cogmed subtasks were included in the final analysis, including “Grid,” “Cube,” “Sort,” “Rotation,” “Twist,” and “3D‐cube.” See also Kolskår et al. (2021) for further details.

### 2.6 Data analysis

All statistical analyses were performed using R, version 3.6.2 (R Core Team, 2019). Group differences were tested using the *t*‐test or Wilcoxon–Mann–Whitney, as appropriate. Before analysis, we imputed 53 missing continuous values of the *n* = 66 dataset, and 43 of the *n* = 52 sample (5% of the total scores were imputed), using the predictive mean matching method from the *mice* package in R (van Buuren & Groothuis‐Oudshoorn, 2011). In addition, all continuous variables were normalized (mean: 0, SD = 1).

The main hypotheses of association between baseline activity level and cognitive training performance and response were addressed using Bayesian linear models as implemented in the *brms* package in R (Bürkner, 2017) interfacing with Stan (Stan Development Team, 2020). Cognitive performance and response were used as dependent variables, and minutes of weekly physical activity in different intensities, and daily minutes of sedentary behavior, were included as independent variables, together with age and sex (model: cognitive performance/‐response ∼ activity level + age + sex). When testing the association between physical activity, and cognitive performance or response, respectively, two and one participants were identified as outliers and potential influential cases, with Cook's distance more than four times below the sample mean. These were removed from the respective analysis. For the same reason, one additional participant was removed from the analysis regarding association between CCT response and minutes of vigorous physical activity. For the analysis of the association between NIHSS and physical activity in chronic phase, activity level was set as dependent variable and NIHSS score as independent variable, in addition to sex and age. Separate models were run for each level of activity intensity (model: activity level ∼ NIHSS + age + sex). Prior probability distributions were set to zero (SD = .5), and the results were presented as mean estimated coefficient, 95% credible interval with the highest density interval (HDI) method, and the Bayes factor (BF) as evidence for null or alternative hypothesis. The Savage–Dickey method (Wagenmakers et al., 2010) was used to calculate BF. For a full presentation of the evidence categories of BF, see Table S1.

Using the Bayesian regression, the question of differences in activity level among three groups defined by the TOAST classification and four groups defined by the location of stroke lesion was addressed. The categories from TOAST and location were coded as multi‐level predictors (model: activity level ∼ 0+ TOAST/stroke lesion location + sex + age). The 0 in the model suppresses the intercepts and estimates the coefficient as the absolute effect of predictors. Prior probability distributions were set to zero (SD = 1) for this analysis. First, each group coefficient (group estimate) from the modeling of the associations between TOAST classification or stroke location and activity/sedentary behavior was extracted. Second, the difference between two and two group coefficients were calculated for TOAST classification and stroke location separately, and the results were presented as mean difference between the group coefficients, 95% credible interval, and directional probability. Directional probability over .5 was an indication of group x > group y and under .5 was an indication of group y > group x.

## 3 RESULTS

### 3.1 Sample characteristics

**FIGURE 2:**
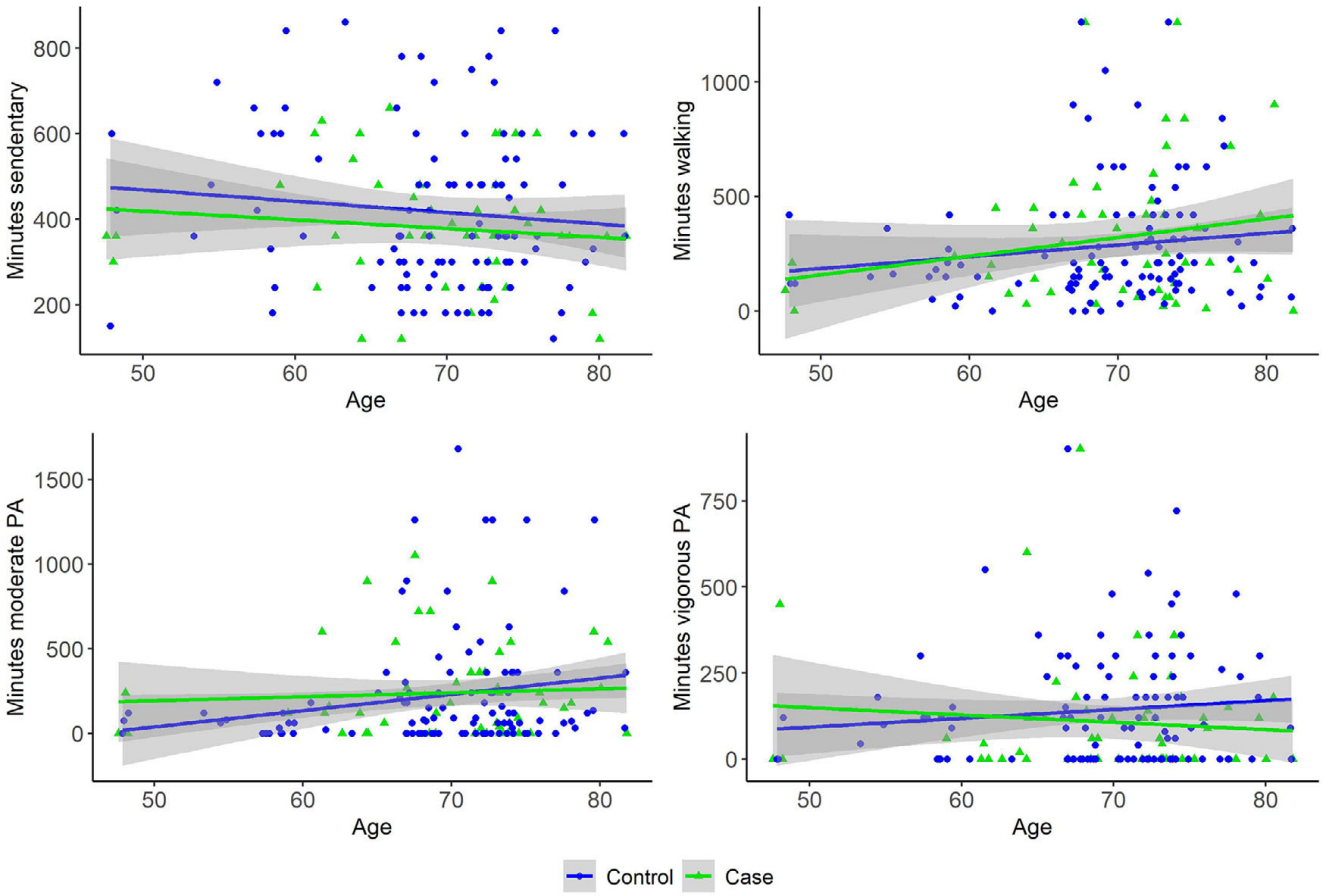
Associations between baseline physical activity (PA) level and age split by case (*n* = 52) and control (*N* = 104). The fit lines represent the linear associations. Bayes factor (control/case) for minutes sedentary = 3.06/2.99, minutes walking = 2.05/1.39, minutes moderate PA = 0.73/3.31, and minutes vigorous PA = 3.00/2.90 (see Table S2 for full overview of estimated coefficients).

Table 1 presents demographic and clinical characteristics for all samples, including case–control comparisons; Figure 2 visualizes the associations between the activity measures and age for both patients and healthy controls; Table S2 presents summary statistics for both models testing the main effect of age and sex on activity level. Briefly, the analyses revealed no evidence in favor of differences in activity levels of various intensities between the longitudinal stroke sample and healthy controls. However, the analysis revealed moderate evidence of a sex difference in daily minutes of sitting in the longitudinal stroke sample (*β* = −.56, HDI = −1.09, −.04, BF = .22), with males being more sedentary (median: 360 [IQR: 120]) compared to females (240 [210]). Anecdotal evidence was found for a positive association between minutes of moderate physical activity and age among healthy controls (*β* = .19, HDI = 0, .38, BF = .73). Median (IQR) NIHSS at discharge from acute stroke unit was 1 (2), ranging from 0 to 7, indicating low‐to‐moderate neurological symptoms.

**TABLE 1:** Sample characteristics.

	Healthy controls		Stroke patients Longitudinal sample (first session)		Case–control comparison^a^	Stroke patients (first session)	
	Mean/Median (SD/IQR)	Min–max	Mean/Median (SD/IQR)	Min–max		Mean/Median (SD/IQR)	Min–Max
**N** *(% female)*	104 (25)		52 (25)		0 (1)^b^	66 (27.3)	
**Age** *[mean (SD)]*	69.1 (7.1)	47.9–81.7	69.5 (7.5)	47.6–81.8	−.31 (.760)^c^	67.9 (10.4)	24.3–81.8
**Education** *[mean (SD)]*	15.8 (3.2)	8–23.5	14.1 (3.8)	7–30	2.69 (.010)^c^	14.1 (3.8)	7–30
**MOCA** *[mean (SD)]*	27 (2.3)	17–30	25.8 (2.8)	17–30	2.75 (.010)^c^	25.6 (3.2)	14–30
**IPAQ** *[median (IQR)*]							
**Low intensity** (minutes/week)	210 (300)	0–1260	210 (340)	0–1260	2633.5 (.790)^d^	210 (360)	0–1260
**Moderate intensity** (minutes/week)	80 (240)	0–1680	155 (360)	0–1050	2440.5 (.320)^d^	170 (360)	0–1080
**Vigorous intensity** (minutes/week)	90 (240)	0–900	52.5 (140)	0–900	3052 (.180)^d^	45 (140)	0–900
**Sitting** (minutes/day)	360 (248)	120–860	360 (158)	120–660	2858.5 (.560)^d^	360 (180)	120–660
**Patient characteristics**							
**Months between stroke and (first session)** *[mean (SD)]*			26 (9)	6–45		27.1 (8.9)	6–45
**NIHSS** *[median (IQR)]*			1 (2)	0–7		1 (2)	0–7
**TOAST** *[n]*		Small‐vessel disease	17			20	
		Cardioembolism	6			7	
		Large‐artery atherosclerosis	20			23	
		Other ethology	3			6	
		Undetermined etiology	5			9	
**Stroke subtype** *[n]*		Ischemic stroke	51			65	
		Intracerebral hemorrhage	1			1	
**Stroke lesion location** *[n]*		Bilateral	4			6	
		Right hemisphere	22			29	
		Left hemisphere	18			22	
		Brain stem/cerebellum	8			9	

Abbreviations: IPAQ, International Physical Activity Questionnaire; MOCA, Montreal Cognitive Assessment; NIHSS, National Institute of Health Stroke Scale; TOAST, Trial of Org 10172 in Acute Stroke Treatment.

^a^ Case–control comparison between longitudinal stroke samples and healthy controls.

^b^ *χ* ^2^ (*p*).

^c^ *t*‐Test (*p*).

^d^ Wilcoxon–Mann–Whitney test (*p*).

### 3.2 Association between activity level, and cognitive training performanceor ‐response

**FIGURE 3:**
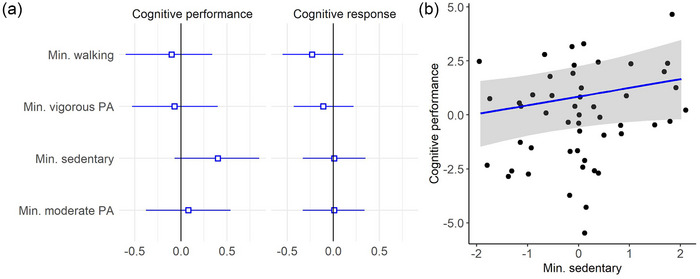
(a) Parameter estimates with 95% credible interval (uncertainty) reflecting the association between cognitive performance and cognitive response with minutes (min.) of physical activity (PA) in various intensities from models additionally adjusted for age and sex. (b) Associations between cognitive performance and daily minutes of sedentary behavior. Points represent observed data, blue regression lines represent estimated slopes, and gray band represents 95% credible interval. Bayes factor = .50. The data was scaled before analysis.

Table 2 presents parameter estimates and corresponding credible intervals for the associations between cognitive performance and outcome from the CCT training, level of physical activity in different intensities, and daily minutes of sedentary behavior. Figure 3 shows the associations between cognitive measures and physical activity, revealing anecdotal evidence of a positive association between cognitive performance and level of sedentary behavior (*β* = .40, HDI = −.07, .85, BF = .50). The analysis showed no other evidence supporting associations between cognitive performance or outcome and physical activity level.

**TABLE 2:** Estimates from the models of the association between cognitive performance and response and activity level, additionally adjusted for age and sex.

Dependent variable	Activity level	Estimate	Lower 95%	Upper 95%	Bayes factor
Cognitive performance	Min. walking	−.10	−.60	.34	1.86
	Min. moderate PA	.08	−.38	.54	1.96
	Min. vigorous PA	−.07	−.53	.40	2.02
	Min. sedentary	.40	−.07	.85	.50
Cognitive response	Min. walking	−.23	−.55	.11	1.15
	Min. moderate PA	.01	−.33	.34	2.94
	Min. vigorous PA	−.11	−.43	.22	2.42
	Min. sedentary	.01	−.33	.35	2.84

Abbreviations: Min., minutes; PA, physical activity.

### 3.3 Association between post‐stroke clinical measures and activity level in chronic phase

Table S3 presents summary statistics for the analysis of the main effect of index stroke characteristics, including three groups of TOAST classification for ischemic stroke etiology and four groups with different locations of stroke lesions on activity level in chronic phase, indicating if each group was associated with increased or reduced activity.

**FIGURE 4:**
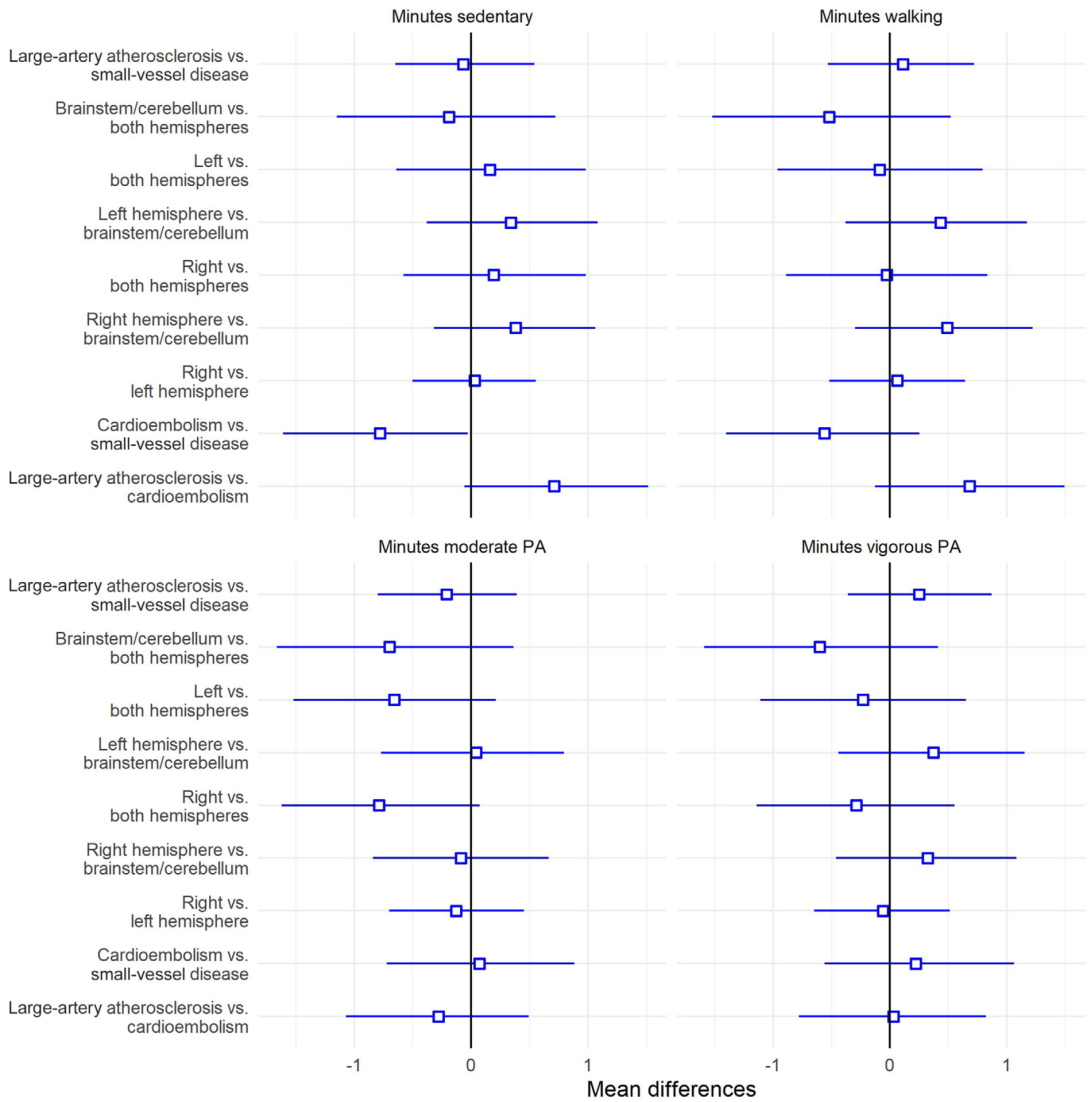
Mean contrast with 95% credible interval (uncertainty) reflecting mean group differences in physical activity (PA) level in various intensities.

Table 3 presents summary statistics from the analysis of contrasts on activity level in chronic phase between different groups with different subtypes and locations of stroke lesions. Figure S1 depicts distribution plot, and Figure 4 shows mean contrasts and credible intervals from the same group analysis. Table 4 presents summary statistics, including parameter estimates and reciprocal credible intervals, for the associations between stroke severity (NIHSS) and level of physical activity in different intensities.

**TABLE 3:** Contrast in effect on activity level between different groups defined by index stroke characteristics.

Activity	Ischemic stroke etiology (TOAST)	Mean difference	Lower 95%	Upper 95%	Directional probability
Min. sedentary	Right vs. left hemisphere	.03	−.50	.55	.55
	Right hemisphere vs. brainstem/cerebellum	.38	−.32	1.06	.86
	Right vs. both hemispheres	.19	−.58	.98	.69
	Left hemisphere vs. brainstem/cerebellum	.34	−.38	1.08	.82
	Left vs. both hemispheres	.16	−.64	.98	.65
	Brainstem/Cerebellum vs. both hemispheres	−.19	−1.15	.72	.34
Min. walking	Right vs. left hemisphere	.06	−.52	.64	.58
	Right hemisphere vs. brainstem/cerebellum	.49	−.30	1.22	.90
	Right vs. both hemispheres	−.03	−.89	.83	.47
	Left hemisphere vs. brainstem/cerebellum	.43	−.38	1.17	.86
	Left vs. both hemispheres	−.09	−.96	.79	.42
	Brainstem/Cerebellum vs. both hemispheres	−.52	−1.52	.52	.16
Min. moderate PA	Right vs. left hemisphere	−.13	−.70	.45	.33
	Right hemisphere vs. brainstem/cerebellum	−.09	−.84	.66	.41
	Right vs. both hemispheres	−.79	−1.62	.07	.03
	Left hemisphere vs. brainstem/cerebellum	.04	−.77	.79	.54
	Left vs. both hemispheres	−.66	−1.52	.21	.07
	Brainstem/Cerebellum vs. both hemispheres	−.70	−1.66		.08
Min. vigorous PA	Right vs. left hemisphere	−.06	−.65	.51	.42
	Right hemisphere vs. brainstem/cerebellum	.32	−.46	1.08	.79
	Right vs. both hemispheres	−.29	−1.14	.55	.25
	Left hemisphere vs. brainstem/cerebellum	.37	−.44	1.15	.83
	Left vs. both hemispheres	−.23	−1.11	.65	.30
	Brainstem/Cerebellum vs. both hemispheres	−.60	−1.59	.41	.12

**Activity**	**Location of stroke lesion**	**Mean difference**	**Lower 95%**	**Upper 95%**	**Directional probability**
Min. sedentary	Large‐artery atherosclerosis vs. cardioembolism	.71	−.06	1.51	.96
	Large‐artery atherosclerosis vs. small‐vessel disease	−.07	−.65	.54	.42
	Cardioembolism vs. small‐vessel disease	−.78	−1.61	−.03	.03
Min. walking	Large‐artery atherosclerosis vs. cardioembolism	.68	−.13	1.49	.95
	Large‐artery atherosclerosis vs. small‐vessel disease	.11	−.53	.72	.64
	Cardioembolism vs. small‐vessel disease	−.56	−1.40	.25	.09
Min. moderate PA	Large‐artery atherosclerosis vs. cardioembolism	−.28	−1.07	.49	.24
	Large‐artery atherosclerosis vs. small‐vessel disease	−.21	−.80	.39	.24
	Cardioembolism vs. small‐vessel disease	.07	−.72	.88	.57
Min. vigorous PA	Large‐artery atherosclerosis vs. cardioembolism	.03	−.78	.82	.53
	Large‐artery atherosclerosis vs. small‐vessel disease	.25	−.36	.87	.79
	Cardioembolism vs. small‐vessel disease	.22	−.56	1.06	.70

Abbreviations: Min, minutes; PA, physical activity; TOAST, Trial of Org 10172 in Acute Stroke Treatment.

**TABLE 4:** Estimates from the models of the association between stroke severity (National Institutes of Health Stroke Scale [NIHSS]) and activity level, additionally adjusted for age and sex.

Clinical variable	Activity level	Estimate	Lower 95%	Upper 95%	Bayes factor
NIHSS	Min. walking	−.10	−.35	.14	3.00
	Min. moderate PA	−.12	−.37	.12	2.47
	Min. vigorous PA	−.16	−.41	.08	1.70
	Min. sedentary	.00	−.23	.22	4.23

Abbreviations: Min., minutes; NIHSS, National Institutes of Health Stroke Scale; PA, physical activity.

Analysis of mean contrast between groups with different subtypes and locations of stroke lesion showed evidence of a difference in daily minutes of sedentary behavior between patients with cardioembolism and patients with small‐vessel disease (mean group difference = −.78, HDI = −1.61, −.03, directional probability = .03), indicating more effect on sedentary behavior of stroke due to small‐vessel disease (*β* = .5, HDI = 0, .99), compared to a cardioembolic stroke (*β* = −.27, HDI = −.97, .42). The analysis showed no other evidence in favor of group differences in clinical measures taken subacutely and activity level in chronic phase.

## 4 DISCUSSION

Cognitive deficits are frequently experienced by stroke patients and represent an important determinant of long‐term outcome and quality of life. Cognitive rehabilitation approaches may improve function, yet the treatment response shows large individual variability. Physical activity has been suggested as a potential actionable factor that may influence the individual response to rehabilitation programs. We tested the association between performance and response on a commonly used CCT system and physical activity level and association between index stroke characteristics, including stroke severity, location, and ischemic stroke etiology, and physical activity level in chronic phase.

The analyses showed anecdotal evidence of a positive association between cognitive performance on the CCT and daily minutes of sedentary behavior, indicating that better cognitive performance was associated with more daily hours of sitting still. We found no support for our hypothesis that patients with higher baseline activity level, both light‐ and moderate‐to‐vigorous physical activity intensity, or a lower number of daily minutes of sitting sill, would show better cognitive performance and more positive effects of CCT. In addition, our additional analysis of the predictive value of index stroke characteristics measured in acute care, including stroke location, ischemic stroke etiology, and stroke severity (neurological symptoms), on physical activity level in chronic phase (>6 months post‐stroke), showed group differences in sedentary behavior between patients with small‐vessel disease and cardioembolism, indicating more sedentary behavior in patients with small‐vessel disease. The analyses revealed no additional associations between index stroke characteristics and physical activity level in this sample of mild‐to‐moderate stroke survivors.

Previous studies have demonstrated negative effects of a sedentary lifestyle on a variety of health marker in adults, including body composition, cardiometabolic risk factors (Saunders et al., 2020; Wirth et al., 2017), and cognitive function. The latter also include working memory (Falck et al., 2017; Kesse‐Guyot et al., 2012). In contradiction to this, a weak positive association between cognitive performance and sedentary behavior was suggested in the present study. One possible explanation for this result can be related to engagement in mentally stimulating activities while sitting. Contrary to watching TV, engaging in regular computer and internet use has been shown to have potentially positive effects on cognitive function in older adults (Hamer & Stamatakis, 2014; Kesse‐Guyot et al., 2012). Although the current study was not conducted on older adults but stroke survivors, it is possible that the participants with higher performance and more time sitting still also engaged in more mentally stimulating activities. Importantly, we did not include an activity log in the current study, allowing us to further explore latent mechanisms behind the association, and there may be other causes of the current finding. There is still need for studies on the effect of computer use on cognition and cognitive training in stroke survivors. Further, the evidence for the association between sedentary behavior and cognitive performance were anecdotal and underpins the need for more research within this field.

Although several studies have suggested a link between cognitive function and moderate‐to‐vigorous physical activity for various populations and ages, albeit with small‐to‐moderate treatment effects and heterogeneous study designs (Erickson et al., 2019; Oberlin et al., 2017), we did not find support for neither an association between cognitive performance and baseline physical activity level of different intensities nor association between response to cognitive training and activity level in the current study. This finding is in line with a recent meta‐analysis of randomized controlled studies on healthy populations, suggesting only small benefits of exercise on cognition and no benefit if correcting for publication biases (Ciria et al., 2023). The current findings underpin the complexity within this field of research and suggest that other factors might be stronger indicators of the performance and success of cognitive training, in chronic phase, than baseline activity level. Future studies are needed to increase the understanding for the role of physical activity in optimizing the potential of cognitive interventions, in both early and late phases post‐stroke.

There is currently a lack of evidence on the prognostic value of index stroke characteristics, measured in acute care, on future physical activity level (Thilarajah et al., 2018), but this information could, for instance, be of relevance in making clinical recommendations on interventions to maintain or increase activity level for specific stroke subgroups or characteristics. Although a previous study suggested group differences in post‐stroke physical activity level between patients with left and right hemisphere strokes (Kunkel et al., 2015), the current findings overall support previous studies showing little predictive value of acute clinical stroke information on physical activity level in chronic phase in mild‐to‐moderate strokes (Wolf & Koster, 2013). Our analysis indicated that patients with small‐vessel disease were more sedentary compared to patients having an ischemic stroke due to cardioembolism. Importantly, inactivity is one of the risk factors for atherosclerosis (Thompson, 2003) and could possibly explain the higher amount of sedentary time in the group with small‐vessel disease. In addition, small‐vessel disease has been linked to executive dysfunction and apathy (Lohner et al., 2017), also offering a possible explanation of the current results.

The current study design ensured a high degree of experimental control over the cognitive training and, as far as possible, securing that each participant received the same intervention. However, some caution should be made when interpreting the results. Our analysis revealed no statistical difference in activity level between stroke survivors and matched healthy controls. Previous studies have shown post‐stroke patients to have about half the number of daily steps in chronic phase compared to matched healthy controls (Fini et al., 2017), suggesting the stroke patients in the current study to be more active than the average stroke patient. Importantly, all participants in the current study were ambulatory, initial stroke severity was mild‐to‐moderate, with a median (min‐max) NIHSS at 1 (0–7), and there was a relatively high drop‐out rate from the intervention, likely due to the required workload. Conceivably, the relatively high demand of the intervention and study procedures may have biased the sample toward a less severe and more high‐functioning part of the overall patient population (Richard, Petersen, et al., 2020; Ulrichsen et al., 2022), influencing the generalizability. Although this high‐functioning group may be more responsive to cognitive interventions and regain function and independence (Eberle & Shapiro‐Rosenbaum, 2022) and therefore are a relevant target in clinical rehabilitation contexts, future studies might benefit from larger samples, including more severely injured patients with more diverse stroke characteristics. In the current study, the level of physical activity was assessed using self‐report. Although commonly used, self‐reporting level of activity is potentially inaccurate due to both underreporting numbers of minutes of light activity and overreporting of high‐intensity physical activity compared to objective measures of physical activity over a longer time period (Prince et al., 2008). The choice to not include sensor‐measured activity level in the current study was based on previous studies showing the potential of some participants being motivated to increase the level of activity due to the monitoring, albeit with small effect sizes (Tayler et al., 2022). Further, previous publications have reported a more positive association between a combination of aerobic and strength training and cognition in stroke survivors compared to aerobic training alone (Oberlin et al., 2017). In the current study, we did not assess the amount of strength training or include aerobic training as an intervention, and we therefore cannot confirm these results. Future studies, including a combination of high‐intensity physical and cognitive training in chronic phase, could yield different results. Lastly, several factors might have influenced the activity level and outcome of the individual patients, beyond index stroke characteristics included in this study. It is still a need for more studies on influential factors, encompassing treatment and clinical follow‐up, as well as biomarkers and brain imaging features, from the acute and sub‐acute phases.

In conclusion, this study of post‐stroke participants in chronic phase, suffering mild‐to‐moderate stroke, revealed overall few associations between level of physical activity, and performanceor response to CCT. The results did not support a the high prognostic value of acute care clinical information on physical activity level in chronic phase. Although the analysis revealed a moderate positive association between cognitive performance and minutes of sedentary behavior and suggested a higher number of minutes of sedentary behavior for patients with small‐vessel disease compared to cardioembolism, other factors beyond post‐stroke physical activity level should be considered in future studies aiming to identify amenable predictors of cognitive performance and response to cognitive rehabilitation in stroke patients.

## AUTHOR CONTRIBUTIONS

**Anne‐Marthe Sanders**: Conceptualization; software; data curation; formal analysis; investigation; visualization; writing—original draft; writing—review and editing. **Geneviève Richard and Kristine M. Ulrichsen**: Conceptualization; investigation; data curation; writing—review and editing. **Knut Kolskår**: Conceptualization; software; data curation; formal analysis; investigation; writing—review and editing. **Dag Alnæs**: Conceptualization; writing—review and editing. **Erlend S. Dørum**: Conceptualization; investigation; writing—review and editing. **Hege Ihle‐Hansen**: Writing—review and editing. **Mads L. Pedersen**: Formal analysis; writing—review and editing; visualization. **Jan Egil Nordvik**: Conceptualization; writing—review and editing; supervision; funding acquisition. **Lars T. Westlye**: Conceptualization; software; supervision; resources; project administration; writing—review and editing; writing—original draft; funding acquisition.

## CONFLICT OF INTEREST STATEMENT

The authors declare that there are no conflicts of interest that could be perceived as prejudicing the impartiality of the research reported.

### PEER REVIEW

The peer review history for this article is available at https://publons.com/publon/10.1002/brb3.3406.

## Supporting information


**Table S1**. Interpretation of Bayes factor (B10) (Modified from Jeffreys, 1961).
**Table S2**. Estimates of the main effect of age and sex on activity level split by group.
**Table S3**. Estimates of the main effect of stroke topography of lesion and TOAST (classification of ischemic stroke) on activity level.
**Figure S1**. Distribution plot of group contrasts in activity level in chronic phase.

## Data Availability

Non‐sensitive data can be made available on request.
