# Supplementary material for: Exploring the associations between physical activity level, cognitive performance, and response to computerized cognitive training among chronic stroke patients
Source: Brain Behav. 2024 Feb 6;14(2):e3406. doi: 10.1002/brb3.3406 (PMC10844996; doi:10.1002/brb3.3406)
Supplement: Supplementary file 1 — Table S1. Interpretation of Bayes factor (B10) (Modified from Jeffreys, 1961). Table S2. Estimates of the main effect of age and sex on activity level split by group. Table S3. Estimates of the main effect of stroke topography of lesion and TOAST (classification of ischemic stroke) on activity level. Figure S1. Distribution plot of group contrasts in activity level in chronic phase. [file BRB3-14-e3406-s001.docx]

**Exploring the associations between physical activity level, cognitive performance and response to computerized cognitive training among chronic stroke patients**

**Authors:** Anne-Marthe Sanders ^a,b,c*^, Geneviève Richard ^a^ , Knut Kolskår ^a,c,d^, Kristine M. Ulrichsen ^a,b,c^, Dag Alnæs ^a^, Erlend S. Dørum ^a,b,c^, Hege Ihle-Hansen ^e^, Mads L. Pedersen ^a,b^, Jan Egil Nordvik ^f,g^ , Lars T. Westlye ^a,b,h^

SUPPLEMENTARY MATERIAL

^a^ NORMENT, Division of Mental Health and Addiction, Oslo University Hospital & Institute of Clinical Medicine, University of Oslo, Norway

^b^ Department of Psychology, University of Oslo, Norway

^c^ Sunnaas Rehabilitation Hospital HT, Nesodden, Norway

^d^ Nordre Aasen Foundation, Kapellveien Habilitation Centre, Norway

^e^ Oslo University Hospital, Oslo, Norway

^f^ Faculty of Health Sciences, Oslo Metropolitan University, Norway

^g^ Norwegian Directorate of Health, Oslo, Norway

^h^ KG Jebsen Center for Neurodevelopmental Disorders, University of Oslo, Oslo, Norway

**Table S1.** Interpretation of Bayes factor (B_10_) (Modified from Jeffreys, 1961).

| **Bayes factor** | **Grade for evidence** | **In favour of hypothesis** |
| --- | --- | --- |
| > 100 | Extreme evidence | H_1_ |
| 30 – 100 | Very strong evidence |  |
| 10 – 30 | Strong evidence |  |
| 3 – 10 | Moderate evidence |  |
| 1 – 3 | Anecdotal evidence |  |
| 1 | No evidence |  |
| 1/3 – 1 | Anecdotal evidence | H_0_ |
| 1/3 – 1/10 | Moderate evidence |  |
| 1/10 – 1/30 | Strong evidence |  |
| 1/30 – 1/100 | Very strong evidence |  |
| < 1/100 | Extreme evidence |  |

**Table S2.** Estimates of the main effect of age and sex on activity level split by group. Model: *Activity level ~ sex + age.*

| **Dependent variable** | **Group** | **Independent variable** | **Mean estimate** | **Lower 95%** | **Upper**  **95%** | **Bayes Factor** |
| --- | --- | --- | --- | --- | --- | --- |
| Minutes sedentary | Control | Age | -0.1 | -0.29 | 0.09 | 3.06 |
|  |  | Sex | -0.09 | -0.51 | 0.30 | 2.17 |
|  | Case | Age | -0.09 | -0.35 | 0.16 | 2.99 |
|  |  | Sex | -0.56 | -1.09 | -0.04 | 0.22 |
| Minutes walking | Control | Age | 0.13 | -0.06 | 0.33 | 2.05 |
|  |  | Sex | -0.04 | -0.45 | 0.38 | 2.39 |
|  | Case | Age | 0.19 | -0.08 | 0.46 | 1.39 |
|  |  | Sex | -0.15 | -0.68 | 0.37 | 1.62 |
| Minutes moderate PA | Control | Age | 0.19 | 0.00 | 0.38 | 0.73 |
|  |  | Sex | -0.01 | -0.41 | 0.40 | 2.49 |
|  | Case | Age | 0.06 | -0.21 | 0.34 | 3.31 |
|  |  | Sex | 0.09 | -0.42 | 0.64 | 1.72 |
| Minutes vigorous PA | Control | Age | 0.10 | -0.09 | 0.28 | 3.00 |
|  |  | Sex | -0.07 | -0.48 | 0.35 | 2.35 |
|  | Case | Age | -0.08 | -0.36 | 0.18 | 2.90 |
|  |  | Sex | -0.08 | -0.64 | 0.44 | 1.68 |

Note. PA, Physical activity.

**Table S3**. Estimates of the main effect of stroke topography of lesion and TOAST (classification of ischemic stroke) on activity level. Model: *Activity level ~ 0 + clinical variable + age + sex.*

| **Dependent variable (activity level)** | **Clinical variable** | **Mean estimate** | **Lower 95%** | **Upper 95%** |
| --- | --- | --- | --- | --- |
| Minutes sedentary | Both hemispheres | 0.14 | -0.57 | 0.88 |
| Minutes walking |  | 0.11 | -0.66 | 0.90 |
| Minutes moderate PA |  | 0.64 | -0.14 | 1.41 |
| Minutes vigorous PA |  | 0.32 | -0.44 | 1.10 |
| Minutes sedentary | Brainstem/cerebellum | -0.05 | -0.63 | 0.57 |
| Minutes walking |  | -0.41 | -1.05 | 0.24 |
| Minutes moderate PA |  | -0.06 | -0.71 | 0.60 |
| Minutes vigorous PA |  | -0.28 | -0.92 | 0.40 |
| Minutes sedentary | Left hemisphere | 0.29 | -0.13 | 0.75 |
| Minutes walking |  | 0.02 | -0.44 | 0.50 |
| Minutes moderate PA |  | -0.03 | -0.50 | 0.45 |
| Minutes vigorous PA |  | 0.09 | -0.40 | 0.56 |
| Minutes sedentary | Right hemisphere | 0.33 | -0.03 | 0.68 |
| Minutes walking |  | 0.08 | -0.31 | 0.49 |
| Minutes moderate PA |  | -0.15 | -0.54 | 0.23 |
| Minutes vigorous PA |  | 0.030 | -0.36 | 0.41 |
| Minutes sedentary | Cardioembolism | -0.27 | -0.97 | 0.42 |
| Minutes walking |  | -0.5 | -1.22 | 0.22 |
| Minutes moderate PA |  | 0.16 | -0.53 | 0.86 |
| Minutes vigorous PA |  | 0.11 | -0.6 | 0.81 |
| Minutes sedentary | Large artery atherosclerosis | 0.44 | 0.03 | 0.83 |
| Minutes walking |  | 0.18 | -0.23 | 0.60 |
| Minutes moderate PA |  | -0.12 | -0.53 | 0.27 |
| Minutes vigorous PA |  | 0.13 | -0.27 | 0.53 |
| Minutes sedentary | Small vessel disease | 0.50 | 0.00 | 0.99 |
| Minutes walking |  | 0.06 | -0.44 | 0.57 |
| Minutes moderate PA |  | 0.09 | -0.39 | 0.57 |
| Minutes vigorous PA |  | -0.11 | -0.61 | 0.39 |

*Note.* PA: Physical activity.

**
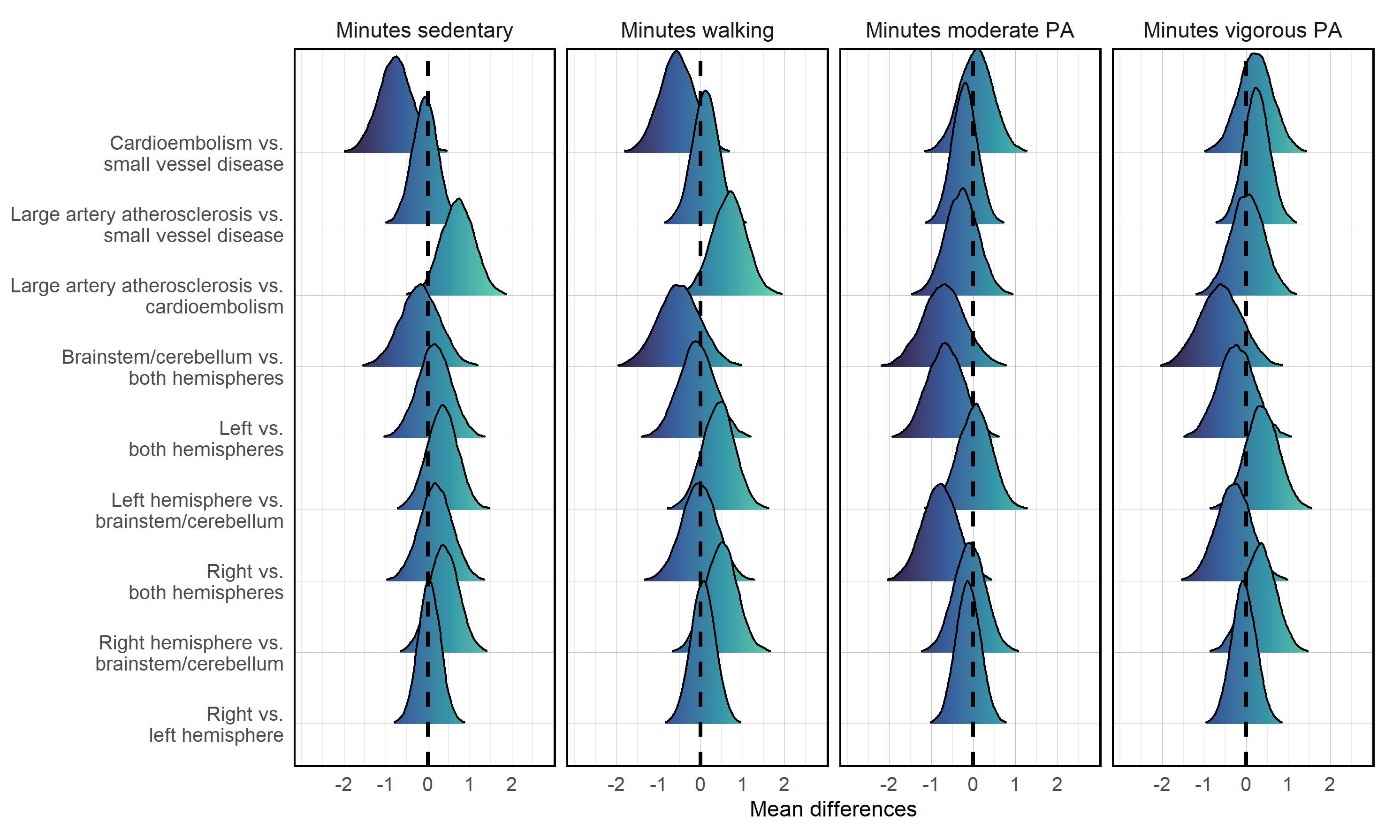
**

**Figure S1.** Distribution plot of group contrasts in activity level in chronic phase. Dotted line represents 0/ no group differences in activity level. The mean of each distribution plot represents the probability for an effect on activity level in first group being larger than the second. The data was scaled before analysis.

**References, Supplementary material:**

Jeffreys, H. (1961). *Theory of probability*, 3rd ed. Oxford: Oxford University Press.
